# Comparison of sexual dysfunction in women with infertility and without infertility referred to Al-Zahra Hospital in 2013-2014

**Published:** 2016-02

**Authors:** Fariba Mirblouk, Dr.Maryam Asgharnia, Robabeh Solimani, Fereshteh Fakor, Fatemeh Salamat, Samaneh Mansoori

**Affiliations:** 1 *Department of Obstetrics and Gynecology, Reproductive Health Research Center, Guilan University of Medical Sciences, Rasht, Iran.*; 2 *Department of Psychiatry, Guilan University of Medical Sciences, Rasht, Iran.*; 3 *Vice-chancellorship for Research, Guilan University of Medical Sciences, Rasht, Iran.*; 4 *Guilan University of Medical Sciences, Rasht, Iran.*

**Keywords:** *Sexual dysfunction*, *Fertility*, *Infertility*

## Abstract

**Background:**

One of the affected aspects in infertile women that have not been given sufficient attention is sexual function. Sexual function is a key factor in physical and marital health, and sexual dysfunction could significantly lower the quality of life. Aim of this study was to assess the comparison sexual dysfunction in women with infertility and without infertility, admitted to Al- Zahra Hospital.

**Objective:**

We decided to assess the prevalence of women sexual disorders in fertile and infertile subjects, admitted to Al-Zahra Hospital.

**Materials and Methods:**

149 fertile and 147 infertile women who referred to infertility clinic of Al-Zahra Hospital during 2013-2014 were entered this cross-sectional study and Female Sexual Function Index questionnaire (FSFI) had been filled by all the cases. Most of women were married for 6-10 years (35.5%) and mean marriage time in participants was 9.55±6.07 years. Data were analyzed using SPSS software Ver. 18 and ^2^ test and logistic regression model has been used for analysis.

**Results:**

Results showed significant differences between desire (p=0.004), arousal (p=0.001), satisfaction (p=0.022) and total sexual dysfunction (p=0.011) in both groups but in lubrication (p=0.266), orgasm (p=0.61) and pain (p=0.793) difference were not significant.

**Conclusion:**

Some of sexual dysfunction indices are high in all infertile women. Our findings suggest that infertility impacts on women’s sexual function in desire, arousal, satisfaction and total sexual dysfunction. Health care professional should be sensitive to impact that diagnosis of infertility can have on women’s sexuality.

## Introduction

Infertility, defined as no pregnancy happening after a year of unprotected sexual intercourse, is a distressing condition affecting about 20% of married couples, with its prevalence having raised in recent years (1-4). The prevalence of primary infertility was 24.9% among 19-49 years old women in a study which has been done in Iran (5). It could weaken the quality of life in both men and women, since mental, physical, social and personal aspects of their lives are under spell (1, 6). 

It will often lead to loss of time and money, marital disruption and divorce in severe situations. The literature demonstrates that women with infertility are at risk of experiencing anxiety, depression and stress, and attempt to suicide are nearly 2 times higher in these cases (1, 6, 7). World Health Organization (WHO) defines sexual health as integrity among mind, emotion, and body which leads the humans’ mental and social dimensions toward improving their personality and eventually results in creation of relationship and love. Therefore, any disorder which disturbs and causes dissatisfaction with sexual relationship can lead to sexual dysfunction (8). 

Sexual function is one of the affected aspects in infertile women which is key factor in physical and marital health, and could decrease the quality of life significantly. Some of sexual dysfunction causes in infertile women are sex hormone and psychological status, duration of marriage, age of patient and negative side effects of medications (9, 10). Sexual complaints are frequent among women, affecting 20-50% of them (11). An American study has demonstrated that these disorders are more common in women (43%) than in men (31%) and are related to demographic characteristics such as age and educational level (6). 40.4% of women in Portugal, and 49% of women in Brazil reported at least one sexual dysfunction (7, 9, 12). Oskay *et al* found that 61.7% of infertile women and 42.9% of fertile women are experiencing sexual dysfunction. In their study, age, partner age and duration of marriage were related with Female Sexual Function Index (FSFI) score (9, 10).

Researchers found that number of people who refer for sexual complaints medical help is much lower than number of people who actually have sexual problems. Women are more likely to ask for professional help than men. Mercer *et al* found that only 21% of women and 11% of men in Great Britain experience more than one sexual complain which have asked for medical care (13, 14). However, physicians mostly do not recognize these concepts during clinical encounter. As a solution, a number of self-assessment questionnaires have been developed over years for evaluating sexual function through a series of sexual domains like desire, arousal, orgasm, or satisfaction (15-19).

During the infertility treatment period, 50-60% of couples reported a considerable reduction in their sexual satisfaction. Losing sexual desire, change in reaching orgasm, reduction of number of intercourses, and sexual dissatisfaction are among common problems experienced by infertile couples (20). In general, sexual problems and disorders are the basic points in evaluation of infertile couples. A desirable sexual relationship can increase probability to conceive. Besides, psychological disorders are assumed to be higher in infertile couples (21, 22). In a study conducted on infertile couples in South Africa, 43% of women believed that inability to conceive had significant negative effects on their lives and particularly sexual relationships (23). Research about this subject in Iran is controversial. Jamali *et al* reported that there was no significant difference between fertile and infertile women regarding their sexual function in Iran (20). 

Direkvand Moghadam *et al* demonstrated that all dimensions of sexual function were lower in infertile women compared with the fertiles (24). Sargolzaei carried out a research in Mashhad which report that sexual dysfunction is more prevalent among the infertile women (25). Despite the importance of these issues to their health care, many women find it hard to talk to physicians about sexual complaints encountered and many physicians find it uncomfortable to discuss such issues with their patients. 

Gynecologists should take the matter more seriously and recommend to their patients to have an evaluation by psychiatrist or psychologist (26, 27). Since the paucity of studies and lack of enough data in Iran and in our province, we decided to assess the comparison of sexual dysfunction in women with infertility and without infertility, admitted to Al-Zahra Hospital, as a significant help to treat infertile women.

## Materials and methods

The study designed as a descriptive cross-sectional study accompanied by a control group, which was approved by the Ethics Committee of Guilan University of Medical Sciences. All participants provided a written informed consent for their participation in the study.

**Participants**

The participants included 17-50 years old infertile and fertile women women admitted to infertility and Gynecology clinic of Al-Zahra Hospital during 2013-2014. 

**Inclusion and exclusion criteria**

In this study all married women aged between 15-70 years old who admitted to Al-Zahra Hospital were included in 2 group of fertile and infertile women. Other criteria were being able to read and write, not suffering from psychological as well as underlying physical disorders, and not having experienced any stressful events during the past 3 months. 

In addition, the fertile women had to have history of at least one delivery and infertile ones had to have either primary or secondary infertility. Patients in infertility group were enrolled in study with documented record of infertility. Cases with not having history of malignancy, a history of administering drugs which cause sexual dysfunctions such as; antipsychotic, benzodiazepines, SSRIS, and other drugs, a history of severe psychological problems like depression, vaginitis and other infections. Male infertility was excluded. 

**Procedure**

Demographic and background data of cases were noted. FSFI questionnaire filled by patients who admitted to clinical center was used. Those who got pregnant without ART and have had at least a 6 months old baby, and were not carrying a baby at the time (with no history of infertility in the husband) considered as healthy fertile women. 

**Instruments**

FSFI questionnaire was used to measure the sexual infertility of cases; FSFI questionnaire include desire for sex, sexual arousal, sexual pleasure, wet vagina, sexual satisfaction and pain. The Farsi version of the questionnaire, in an evaluation accomplished by Mohammadi *et al*, has been reported as an appropriate mean to screen disorders in this field. This questionnaire has good reliability and validity that was evaluated by Mohammadi *et al* in Iran. The reliability of questionnaire was reported as 78% and 75% using split-half and test-retest methods, respectively. In addition, reliability of subscales was between 63% and 75% through split-half and between 70% and 81% through test-retest method (28). The questionnaire had been filled by all fertile and infertile women.

According to instruction of questionnaire designer about scoring, each section’s score has counted by summing the scores of each section’s question multiplied in factor’s number (since in FSFI questionnaire, the total number of question in each section are not the same, to equiponderate the section to each other, first the scores of each section’s questions summed up together, and then multiplied in the factor’s number). Scores considered in desire section defined as 1-5 and the sexual arousal section, orgasm and pain was defined as 0-5 or 1-5. Null describes that the person has not had sexual intercourse in last 4 weeks. By summing up the scores of each section, we reach the total evaluation score. 

The higher score considered the healthier sexual activity. According to sections equiponderate, the maximum score of each section defined as 6 and for the whole evaluation as 36. The minimum score in desire section will be 2.1, in sexual arousal, wet vagina, pain and orgasm is 0, and in satisfaction section is 0.8 and for the whole evaluation is 2. Score of each section less than 65% of the maximum (<3.9) defined as disability in that section and total score less than 28 defined as total sexual inability (29). 

**Studies limits**

There were a few cooperation problems with some patients. Access to other private hospitals in Rasht for better sampling was another limitation of study. 

**Statistical analysis**

The Chi Square test has been used to adjust the variables in fertile and infertile groups, and to calculate the matched odds ratio of variable for infertility. We used the logistic regression model (step wise). We judged a p<0.05 as significant.

## Results

The FSFI questionnaire had been filled by all 149 fertile and 147 infertile women who referred to Gynecology and infertility clinic of Al-Zahra Hospital during 2013-2014. All participants respond to questionnaire. Most of women referred to Al-Zahra hospital were married for 6-10 years (35.5%).The mean age was 31.66±6.8 years old and mean length of marriage was 9.55±6.07 years. There was no significant difference between two groups in demographic data except for the age (p=0.001) and marriage duration (p=0.008). Table I indicates the clinical and demographic data of the cases.

Disorders in desire in fertile group have been reported as 54.4% and in infertile group as 69.8% (p=0.006), while disorder in arousal in fertile group was 47.6% and in infertile group 63.8% (p=0.005), disorder in orgasm in fertile group was 13.6% and in infertile group 23.5% (p=0.03). Table II showed mean of sexual dysfunction subscales and total sexual dysfunction. These results showed significant differences between desire (3.93±0.94 vs. 3.62±0.91, p=0.004), arousal (4.12±0.90 vs. 3.71±1.06, p=0.001), satisfaction (4.99±0.87 vs. 4.74±1.00, p=0.022) and total sexual dysfunction (26.33±3.82 vs. 34.40±25.13 p=0.011) in infertile and fertile groups but in lubrication (p=0.266), orgasm (p=0.61) and pain (p=0.793) were not significant. Marital history evaluation demonstrated that the prevalence of sexual dysfunctions arise with increasing the marriage duration. 

The rate of sexual dysfunctions were 3 times higher than couples married for 6-10 years compare with couples who were married for less than 5 years, also in couple who were married for more than 10 years, the prevalence were 12 (Table III).

**Table I:** Demographic characteristics of study units

**Variable**		**Infertile (n=149)**	**Fertile (n=147)**	**p-value**
Marriage duration		6.75 ± 4.27	12.31 ± 6.33	0.001
Age		30.61 ± 6.67	32.71 ± 6.79	0.008
Duration of marriage				
	<5 years	74 (50.3)	20 (13.4)	0.001**
	6-10 years	51 (34.7)	54 (36.2)	
	>10 years	22 (15)	75 (50.3)	
Job				
	Home keeper	127 (86.4)	134 (89.9)	0.35
	Employed	20 (13.6)	15 (10.1)	
Age group				
	≤18	1 (0.7)	0	0.07
	18-25	35 (23.8)	23 (15.4)	
	25-35	80 (54.4)	78 (52.3)	
	>35	31 (21.1)	48 (32.2)	
Body mass index				
	<19	4 (2.7)	3 (2.0)	0.83
	19-25	49 (33.3)	51 (34.2)	
	25-30	62 (42.2)	68 (45.6)	
	>30	32 (21.8)	27 (18.1)	
Education				
	Primary	68 (45.6)	52 (35.4)	0.13
	Diploma	62 (41.6)	67 (45.6)	
	Post diploma	19 (12.8)	28 (19)	

* data are presented as frequency (%) or Mean ±SD.

** significant

**Table II:** Comparison of sexual dysfunction in different dimensions in fertile and infertile women

**Sexual dysfunction dimensions**	**Infertile (n=147)**	**Fertile (n=149)**	**p-value**
Desire	3.93 ± 0.94	3.62 ± 0.91	0.004*
Arousal	4.12 ± 0.90	3.71 ± 1.06	0.001*
Lubrication	4.76 ± 0.88	4.64 ± 0.95	0.266
Orgasm	4.67 ± 0.89	4.45 ± 1.10	0.61
Satisfaction	4.99 ± 0.87	4.74 ± 1.00	0.022*
Pain	3.86 ± 1.09	3.90 ± 1.21	0.793
Total sexual dysfunction	26.33 ± 3.82	34.40 ± 25.13	0.011*

* data are presented as Mean±S.D.

** significant

**Table III:** The adjusted OR for infertility

**Variable**		**Adjusted OR**	**CI (95%)**	**p-value**
Duration of marriage				
	<5 years	1	-	
	6-10 years	3.85	(2.05-7.23)	0.001
	>10 years	12.03	(6.04-23.98)	0.001
Arousal dysfunction		1.7	(1.01-2.86)	0.04

OR: odds ratio

CI: Confidence Interval

## Discussion

Infertility has different effects on various dimensions of women’s life, such as sexual relationship. Sexual satisfaction is mainly affected by outcomes of infertility (20). Lack of sexual satisfaction caused by many psychological disturbances and marital discords (24). Sexual behaviors are complex and different based on individual interactions with others, cultural and life status. This behavior has close relation with biological structure and self-esteem, experiences and individual changes about sexual issues (30). 

In present study we tried to evaluate the comparison between women sexual patterns and sexual dysfunctions in two groups of women with and without infertility. Results showed that there was significant difference between two groups in most of subscales and total sexual dysfunction score. The prevalence of female sexual dysfunction (FSD) was 74.5% among Iranian infertile women in this study. This figure is a little different from those found in other epidemiological studies assessing FSD in infertile women. Studies conducted in the US (n=218) and in Iran found a lower prevalence of sexual dysfunctions (40 and 48%, respectively) (5, 31). 

Pakpour *et al* evaluated the prevalence of sexual dysfunction in women . 636 Iranian infertile women had been assessed, which the prevalence of sexual dysfunction among infertile women has been reported as 56%. The reason of difference can be related to age differences of participating women, age range was 17-50 years in our study while it was 19-45 years in Pakpour study (32). Factors such as age, duration of marriage and partner age have been considered as main causes of sexual complaints in infertile women. In the current study, longitude of marriage was significantly different between two groups (p=0.001). Similar to Oskay *et al* study we also found correlation between total FSFI score and marriage duration (33).

Regarding BMI score, our results are consistent with those of Keshin *et al* (34). Both studies demonstrated that BMI was not statistically different between the infertile groups. Regression analysis demonstrated that age is not an independent predictor of sexual function in women suffering from infertility in our study. In a study conducted by Keshin *et al* age and income were independent predictors of FSFI score (34). We include all variables with p<0.25 in logistic regression (stepwise) model as independent factor to adjust the variables. Risk of infertility demonstrated an obvious increase in association with longitude of marriage and arousal dysfunction. (Hosmer and Lemeshow Test, p=0.995). The findings showed that there was no significant difference in women’s sexual dysfunction between groups.

In our study the most common alterations were lack of desire, arousal and orgasm. Desire and arousal disorders are among the most common complaints in clinical practice, because there is a high correlation between both (28). Nevertheless, in a study on Brazilian population, the most common disorders are the absence of orgasm and lack of sexual desire, which is considered the most frequent sexual disorder in women which is consistent with the results of Monga *et al*; they assessed 12 fertile and 18 infertile couples, and reports no significant difference in women’s sexual function scores between groups, whereas the quality of life in infertile group was effectively lower than in fertile group (7, 30). 

These results are against the results of Oskay *et al* which total FSFI score and score of each domain were significantly higher in control group than infertile group, the total FSFI score was 24.58 in infertile group and 26.55 in control group (33). In another study, Millheiser *et al* evaluated infertile and fertile women and reported sexual dysfunction in 40% of infertile and 25% of healthy control women (31). In their study, scores for desire, arousal and satisfaction domains of FSFI questionnaire were significantly higher in healthy controls, whereas in our study scores of all domains were statistically higher in controls (31). By means of Sexual Function Questionnaire, Khademi *et al* reported arousal and orgasm dysfunction in up to 80% and 22% of infertile women, respectively (13). 

There are several studies in line with our results which reported decreased the sexual function in infertile women (20, 35, 36). Jamali *et al* reported a significant decrease of sexual satisfaction in infertile women (20). In another study sexual satisfaction and sexual function were examined in infertile women. The results of study showed that infertile women have significant decrease in all domains of their function and sexual satisfaction (36). In same line, Noorani *et al* conducted a study in Mashhad in 2008 and showed that sexual satisfaction was insignificantly higher among infertile women (37). Sattarzadeh *et al* showed that infertile women’s sexual function mean score was different from fertile women, but difference was not statistically significant (38). 

**Limitations**

First, we did not collect comprehensive information of control group. Second, we have not evaluated the reason of patients who presented the hospital. It could be disease or anything that has an effect on sexual function on women. Also, men’s sexual functions may have effects on women’s, so the evaluation of men’s sexual functioning is recommended. We had little information about women’s partners. 

## Conclusion

Sexual dysfunction is high in all infertile women. Our findings suggest that infertility impacts women’s sexual function. Health care professional should be sensitive to impact that diagnosis of infertility can have on women’s sexuality. Our findings suggest that infertility impacts on women’s sexual function in desire, arousal, satisfaction and total sexual dysfunction in women with infertility without infertility. Although our results showed a unique pattern in this region which can be related to other factors. Multi-center investigations with larger sample sizes, comprehensive evaluation of male partners and control group in future studies in this region is recommended.

## Note

***This article extracted from M.D. thesis. (Samaneh Mansoori)***
